# Inflammatory and Angiogenic Factors at Mid-Pregnancy Are Associated with Spontaneous Preterm Birth in a Cohort of Tanzanian Women

**DOI:** 10.1371/journal.pone.0134619

**Published:** 2015-08-06

**Authors:** Chloe R. McDonald, Anne M. Darling, Andrea L. Conroy, Vanessa Tran, Ana Cabrera, W. Conrad Liles, Molin Wang, Said Aboud, Willy Urassa, Wafaie W. Fawzi, Kevin C. Kain

**Affiliations:** 1 SAR Laboratories, Sandra Rotman Centre for Global Health, University Health Network-Toronto General Hospital, University of Toronto, Toronto, Canada; 2 Institute of Medical Science, University of Toronto, Toronto, Canada; 3 Department of Global Health and Population, Harvard School of Public Health, Boston, Massachusetts, United States of America; 4 Department of Nutrition, Harvard School of Public Health, Boston, Massachusetts, United States of America; 5 Department of Medicine, University of Washington, Seattle, Washington, United States of America; 6 Department of Epidemiology, Harvard School of Public Health, Boston, Massachusetts, United States of America; 7 Department of Microbiology and Immunology, Muhimbili University of Health and Allied Sciences, Dar es Salaam, Tanzania; 8 Tropical Disease Unit, Division of Infectious Diseases, Department of Medicine, University of Toronto, Toronto, Canada; University of Cape Town, SOUTH AFRICA

## Abstract

**Research Question:**

Preterm birth (PTB) is the leading cause of perinatal mortality worldwide, with the greatest burden occurring in resource-constrained settings. Based on the hypothesis that altered placental angiogenesis and inflammation early in pregnancy lead to PTB, we examined whether levels of inflammatory and angiogenic mediators, measured early in pregnancy, were predictive of spontaneous PTB (sPTB).

**Study Design:**

Plasma samples were collected from a prospective cohort of primigravid Tanzanian women between 12–27 weeks gestation. A panel of 18 markers was screened on a training cohort of 426 women. Markers associated with sPTB in the training cohort were repeated in a test cohort of 628 women. All markers were measured by ELISA.

**Findings:**

In both the training and test cohorts plasma levels of IL-18BP, sICAM-1, sEndoglin and CHI3L1 were elevated and Leptin was lower at enrollment in women who subsequently experienced sPTB. In multivariate analysis women with plasma levels of CHI3L1, C5a, sICAM-1, AngptL3, sEndgolin, sFlt-1 and IL-18BP in the highest quartile had an increased risk of sPTB compared with those in the lowest quartile. Women with Leptin and Ang2 in the highest quartile had a reduced risk of sPTB compared with women in the lowest quartile.

**Implications:**

Levels of angiogenic and inflammatory mediators measured at mid-pregnancy were associated with subsequent sPTB. These findings provide insight into mechanisms underlying sPTB and suggest biomarkers that may have clinical utility in risk-stratifying pregnancies.

## Introduction

Preterm birth (delivery <37 weeks gestation) is the leading cause of perinatal mortality worldwide [1, 2]. Despite the introduction of public health and intervention strategies, rates of preterm birth (PTB) are rising in both developed and developing countries [3, 4]. It is estimated that each year 14.9 million births are preterm with the majority occurring in resource-constrained settings [1, 2]. In addition to its impact on mortality, PTB is associated with long term disabilities in survivors including neurodevelopmental disorders and an increased risk of chronic diseases [5]. The financial and social costs associated with PTB place a large burden on families and public health systems, particularly in low-resource settings [6, 7].

Despite its impact on global health, little is known about the mechanisms underlying spontaneous PTB (sPTB). In high resource settings an estimated 70% of all prematurity is due to sPTB [8]. This rate is likely higher in resource-constrained settings where medically indicated or elected preterm delivery is rare [9]. Current evidence suggests that the events leading to sPTB occur early in pregnancy and are linked to the premature initiation of inflammatory pathways and alterations in what are normally tightly regulated angiogenic processes [10–12]. While the induction of inflammatory pathways play a role in the initiation of labor at term [12, 13], clinically apparent and sub-clinical infections may initiate these pathways prematurely, inducing PTB [13, 14]. Inflammation has also been observed in PTB in the absence of infection, suggesting inflammation may be a common pathway leading to premature birth [15].

Disruptions to normal angiogenic processes early in pregnancy may also contribute to sPTB by altering the balance between pro- and anti-angiogenic factors required for normal placental vascular remodeling and fetal growth [16]. Histopathological evidence of abnormal placentation has been reported in cases of spontaneous preterm delivery [17, 18]. Altered placentation, insufficient vascular development and disruptions to the normal transformation of spinal arteries can lead to increased placental vascular resistance coupled with reduced blood flow in the intervillous space [18, 19]. The resulting limited nutrient delivery to the fetus may ultimately contribute to sPTB due to the inability of the placenta to support third trimester fetal growth [18, 20].

In this study we used a train and test study design to test the hypothesis that biomarkers of angiogenic and inflammatory pathways measured at mid-pregnancy may identify women at risk of sPTB in a low resource setting. Biomarkers of sPTB could help elucidate the mechanisms underlying prematurity, develop predictive tools to identify high-risk pregnancies, and identify new targets for intervention for this global health priority.

## Materials and Methods

### Study Site and Participants

This study cohort was nested within a larger prospective trial of multivitamin supplementation in pregnant women conducted in Dar es Salaam, Tanzania [21]. In this randomized trial, HIV negative women with an estimated gestational age (based on last menstrual period) between 12 and 27 weeks attending antenatal clinics in Dar es Salaam were invited to participate. Enrollment blood samples were collected (one sample per participant) prior to assignment to treatment arms (daily micronutrient supplementation or placebo). All women, regardless of treatment arm, were given daily doses of iron (60 mg of elemental iron) and folic acid (0.25 mg) as well as presumptive anti-malarial therapy with sulfadoxine-pyrimethamine tablets (Fansidar, Roche) at 20 and 30 weeks gestation as described [21]. In the present study samples were tested separately at two distinct times and in two testing cohorts; first a training cohort (assays conducted in January 2012) and second, a test cohort (assays performed in January 2013). The train and test approach enables internal validation of the biomarkers using assessment of the biomarker panel in a training cohort followed by refinement and validation of the panel in the test cohort.

### Training Cohort

The training cohort was comprised of primigravid women with singleton live birth and known birth outcome (n = 432) that were randomly selected from this larger prospective study (N = 8648)[21]. Random sample selection was achieved through the use of a random number list generated by SAS v9.2 (SAS Institute Inc., Cary, NC) software. Sample size (n = 432) was calculated based on previous data indicating the power required to detect clinically significant differences in mean angiopoietin-1 (Ang1) and Ang2 levels in peripheral plasma that were associated with adverse birth outcomes[22]. All cases of induced PTB (n = 6) were excluded.

### Test Cohort

The test cohort consisted of all remaining primigravid women with singleton live birth and known birth outcome (n = 646) from the parent trial (N = 8648) that fit the inclusion criteria. All cases of induced preterm birth were excluded (n = 18). From the combined cohort (training plus test sets) a total of 1054 samples were analyzed.

### Ethics Statement

Ethical approval for this study was obtained from review boards at the Muhimbili University College of Health and Allied Sciences, the Harvard School of Public Health and the University Health Network. Signed consent forms were obtained from all participants in the initial cohort study to allow for blood collection and subsequent protein analysis of plasma samples.

### Biomarker Assays

Maternal peripheral plasma samples were collected and stored in EDTA at -80°C prior to testing. Plasma samples were assessed (as indicated) for levels of Ang1, Ang2, Angiopoietin-Like 3 (AngptL3), Vascular Endothelial Growth Factor (VEGF-A), Factor D, Monocyte Chemoattractant Protein-1 (MCP-1), Interleukin-1 Beta (IL1B), Soluble fms-like tyrosine kinase 1 (sFlt-1), soluble Tumor Necrosis Factor Receptor 2 (sTNFR2), Placental Growth Factor (PGF), Macrophage Inflammatory Protein-1 Beta (MIP-1β), Leptin, Interleukin-18 Binding Protein (IL-18BP), soluble Intercellular Adhesion Molecule-1 (sICAM-1), soluble Endoglin (sEndoglin), C-reactive protein (CRP), Chitinase-3-Like Protein-1 (CHI3L1), and complement component C5a (C5a) using commercially available enzyme linked immunosorbent assay (ELISA) kits (Duosets, R&D Systems, Minneapolis, MN). All laboratory procedures followed the manufacturers’ protocol (R&D System Duosets) with two notable exceptions; our laboratory used a longer incubation period (overnight at 4°C) and samples were developed using ExtrAvidin (1:1000 dilution, Sigma-Aldrich Co.) and SIGMA*FAST* p-Nitrophenyl phosphate Tablets (Sigma-Aldrich Co.). Sample dilutions were determined based on previous pilot studies and results in our laboratory. Analysis was performed blinded to the patient group. Biomarkers were selected based on previous studies implicating these factors, or the pathways they reflect, as potential mediators of adverse pregnancy outcomes [12–16, 19, 20]

### Statistical Analysis

Statistical analysis was performed using SAS v9.2 (SAS Institute Inc., Cary, NC), STATA v12.1 (StataCorp., College Station, TX), SPSS v20 (IBM Corp., Armonk, NY) and GraphPad Prism v5 (GraphPad Software Inc., La Jolla, CA) software. Baseline characteristics were compared between women who delivered sPTB and women who delivered at term. Categorical variables were compared using the Pearson Chi-square test or Fisher’s exact test where appropriate. Concentrations of biomarkers showed deviation from normality (Shapiro-Wilks p < 0.05), and therefore were non-parametrically tested for differences in biomarker levels between groups using the Wilcoxon rank-sum test. We controlled for multiple comparisons using a Bonferroni-corrected p-value. Since the balance of pro and anti-angiogenic mediators required for successful birth outcomes is tightly regulated across gestation, polynomial curves with the best fits to the data were used to examine the effect trends in peripheral biomarker levels in relation to gestational age at enrollment. We compared first order polynomial and second order polynomial curves using the least squares fitting method. The best model was selected based on F-test.

Log binomial regression with the log link function was used to estimate relative risks of sPTB and 95% confidence intervals across quartiles of biomarker levels, with the lowest quartile as the reference category. When the model failed to converge, a log-Poisson model was used. Baseline variables associated with the primary outcome of interest, spontaneous preterm delivery, at a p-value less than 0.2 were included in the multivariate model. Multivariate models were adjusted for maternal age (continuous), marital status (yes/no), education (0–4, 5–7, 8–11, ≥ 12 years), Filmer-Pritchett wealth score less than median (yes/no), baseline gestational age (continuous), body mass index (BMI: kg/m^2^) at baseline (<18.5, 18.5–24.9, ≥25), and frequency of animal protein consumption per week (once a week or less vs. more than once per week). Micronutrient supplementation was associated with reduced incidence of low birth weight and small-for-gestational age outcomes had no impact on PTB in the randomized trial [21] and therefore it was not included in the multivariate analysis. Missing indicator variables, mean values, were imputed to replace missing observations (11.2%) for BMI. We tested for the presence of linear trends by assigning each quartile the median value and modeling this variable as a continuous variable.

## Results

### Characteristics of Study Population

The baseline characteristics of the train and test cohorts are shown in S1, S2 and S3 Tables. There were no significant differences between baseline characteristics of women in training and test sets (S1 Table). The baseline characteristics of the combined cohort are reported in Table 1. In the total study population of 1054 primigravid women, there were 156 spontaneous preterm deliveries (57 occurring in the training cohort and 105 in the test group). Overall women who delivered sPTB had a lower gestational age at enrollment, lower maternal age, were more likely to be unmarried, had fewer years of education, lower BMI and skinfold thickness, lower birth weight, and lower socio-economic status (as indicated by Filmer-Pritchett wealth score). The median gestational age [IQR] at delivery (combined training and test cohorts) in women who delivered at term was 40.1 [39.0, 41.4] weeks compared with 34.7 [33.2, 36.1] weeks for women who spontaneously delivered preterm.

**Table 1:** Descriptive characteristics of study cohort.

	Birth Outcome	
Variable	Term (n = 892)	sPTB (n = 162)
**Gestational age at enrollment (weeks)**	22 [19.3, 24.4]	19.9 [17.1, 22.4]
**Gestational age at delivery (weeks)**	40.1 [39.0, 41.4]	34.7 [33.2, 36.1]
**Maternal age (years)**	21.5 [19.5, 23.5]	20.5 [18.5, 22.5]
**Education (years)**		
0–4	68 (7.5)	17 (10.8)
5–7	596 (65.9)	109 (69.4)
8–11	196 (21.7)	27 (17.2)
≥ 12	44 (4.9)	4 (2.5)
**Marital status**		
Married	183 (20.2)	114 (72.6)
Divorced/single/widowed	72 (79.4)	42 (26.8)
**Body Mass Index (kg/m** ^**2**^ **)**	23.4 [21.5, 25.6]	23.1 [21.3, 25.2]
**Baseline Hemoglobin (g/dL)**	10.2 [9.2, 11.1]	9.9 [8.7, 10.8]
**Baseline Skin-fold Thickness (cm)**	17.1 [13.1, 21.6]	14.9 [11.8, 18.5]
**Birth Weight (g)**	3000 [2800, 3400]	2900 [2500, 3100]
**Peripheral Malaria Parasitaemia**		
Yes	6 (0.7)	2 (1.3)
No	898 (99.3)	155 (98.7)
**Literacy**		
Yes	821 (90.8)	140 (89.2)
No	80 (8.8)	17 (10.8)
**Frequency of meat/fish consumption**		
< 1x per week	60 (6.6)	12 (7.6)
≥ 1x per week	844 (93.3)	145 (92.4)

Median [IQR] or number (%) where applicable

Altered circulating levels of angiogenic and inflammatory factors at mid-pregnancy are associated with spontaneous PTB in the training cohort

In the training cohort (n = 426), primigravid women who went on to deliver sPTB had increased circulating levels of IL-18BP (*P* = 0.01), sICAM-1 (*P* = 0.04), sEndoglin (*P* = 0.0002), CHI3L1 (*P* = 0.008) and decreased Leptin (*P* = 0.03), compared with women who delivered at term (Table 2). Elevations in levels of sEndoglin were statistically significant after controlling for multiple comparisons (*P*<0.003).

**Table 2:** Median biomarker^a^ levels (pg/mL) according to term and preterm birth status in the training cohort.

Biomarker^a^	Term Birth		Pre Term Birth		
	n^b^	Median [IQR]	n^b^	Median [IQR]	P-Value^c^
**Ang-1**	368	19.43 [12.03, 28.83]	57	18.82 [9.83, 29.29]	0.40
**Ang-2**	368	4.65 [2.10, 8.85]	57	3.93 [1.32, 8.16]	0.22
**AngptL3**	369	120.01 [82.39, 167.15]	57	127.21 [88.52, 157.70]	0.43
**VEGF**	368	51.08 [7.81, 320.28]	57	69.54 [7.81, 280.93]	0.74
**sFLT-1**	369	1.18 [0.49, 3.80]	57	1.85 [0.95, 3.99]	0.08
**sTNFR2**	369	5.45 [3.90, 7.99]	57	6.42 [4.21, 8.40]	0.16
**PGF**	350	1.42 [0.81, 2.49]	55	1.83 [0.97, 3.60]	0.06
**MIP-1β**	357	152.45 [73.64, 337.73]	55	145.38 [92.74, 355.40]	0.68
**MCP-1**	360	46.68 [9.94, 254.69]	56	43.86 [13.59, 199.03]	0.93
**Leptin**	368	8.00 [4.69, 12.86]	57	6.60 [3.83, 10.17]	0.03
**IL-1β**	368	20.56 [3.91, 76.64]	57	14.07 [3.91, 57.56]	0.25
**IL-18 BP**	369	13.07 [9.10, 18.09]	57	14.97 [11.36, 21.98]	0.01
**sICAM-1**	369	155.65 [111.65, 228.33]	57	196.09 [132.56, 273.68]	0.04
**Factor D**	360	489.88 [330.27, 675.78]	56	469.14 [319.52, 617.41]	0.53
**sEndoglin**	368	22.26 [15.49, 29.89]	57	27.18 [20.56, 39.96]	0.0002
**CRP** ^d^	353	1.83 [0.78, 4.15]	56	2.21 [0.88, 4.58]	0.41
**CHI3L1**	353	37.42 [21.87, 63.74]	57	51.57 [32.23,104.56]	0.008
**C5a**	357	32.20 [17.24, 62.98]	56	36.53 [16.97, 67.46]	0.65

^a^Angiopoietin-1 (Ang1), Angiopoietin-2 (Ang2), Angiopoietin-Like 3 (AngptL3), Vascular Endothelial Growth Factor (VEGF), Soluble fms-like tyrosine kinase 1 (sFlt-1), soluble Tumor Necrosis Factor Receptor 2 (sTNFR2), Placental Growth Factor (PGF), Macrophage Inflammatory Protein-1 Beta (MIP-1β), Leptin, Interleukin-18 Binding Protein (IL-18BP), soluble Intercellular Adhesion Molecule-1 (sICAM-1), soluble Endoglin (sEndoglin), C-reactive protein (CRP), Chitinase-3-Like Protein-1 (CHI3L1), and complement component C5a (C5a)

^b^ Sample sizes were not equal for all biomarkers due to indeterminate assay results, where samples with undetectable values were excluded.

^c^ Results of Wilcoxon rank-sum test.

^d^ Values in mg/mL.

To account for socioeconomic and clinical risk factors of PTB observed between groups, multivariate models were developed adjusting for age, marital status, education, wealth score, BMI and gestational age at enrollment. After adjustment, women with levels in the highest quartiles for PGF, sICAM-1 and sEndoglin had an increased risk of delivering pre-term compared to women in the lowest quartile. Women with PGF and sEndoglin in the highest quartile were over two times more likely to deliver preterm compared with women in the lowest quartile. Women in the highest quartile for Ang2 and Leptin had a minimum 54% reduced risk of sPTB (p < 0.05, Table 3). There was evidence of a linear trend in the associations between each biomarker and sPTB (p-value <0.05 for linear trend test).

**Table 3:** Multivariate relative risks (95% Confidence Intervals) for sPTB according to quartiles in the training cohort^a^ ^,^ ^b^.

	Q1	Q2	Q3	Q4	P-trend^c^
**Ang-1**	1.00 (Ref)	0.58 (0.29, 1.14)	0.74 (0.40, 1.48)	0.98 (0.52, 1.85)	0.77
**Ang-2**	1.00 (Ref)	0.68 (0.38, 1.22)	0.58 (0.29, 1.17)	0.38 (0.19, 0.78)	0.01
**AngptL3**	1.00 (Ref)	1.87 (0.89, 3.93)	2.38 (1.17, 4.85)	1.52 (0.68, 3.43)	0.25
**VEGF**	1.00 (Ref)	1.27 (0.62, 2.62)	1.26 (0.65, 2.43)	1.42 (0.71, 2.84)	0.44
**sFLT-1**	1.00 (Ref)	0.95 (0.42, 2.15)	1.96 (0.96, 4.00)	1.32 (0.62, 2.83)	0.58
**sTNFR2**	1.00 (Ref)	0.62 (0.26, 1.46)	1.67 (0.85, 3.26)	1.11 (0.57, 2.16)	0.33
**PGF**	1.00 (Ref)	1.39 (0.64, 2.99)	1.70 (0.77, 3.75)	2.38 (1.12, 5.07)	0.02
**MIPB**	1.00 (Ref)	1.56 (0.74, 3.27)	0.90 (0.40, 2.01)	1.24 (0.57, 2.69)	0.92
**MCP-1**	1.00 (Ref)	1.33 (0.67, 2.64)	1.14 (0.55, 2.36)	1.01 (0.49, 2.10)	0.64
**Leptin**	1.00 (Ref)	0.85 (0.46, 1.54)	0.48 (0.23, 1.03)	0.53 (0.26, 1.08)	0.06
**IL-1β**	1.00 (Ref)	0.73 (0.38, 1.40)	0.62 (0.30, 1.26)	0.83 (0.47, 1.49)	0.99
**IL-18 BP**	1.00 (Ref)	1.31 (0.59, 2.92)	1.64 (0.76, 3.57)	1.84 (0.87, 3.89)	0.09
**sICAM-1**	1.00 (Ref)	0.79 (0.34, 1.83)	1.17 (0.55, 2.51)	1.84 (0.96, 3.51)	0.009
**Factor D**	1.00 (Ref)	0.87 (0.44, 1.70)	1.01 (0.52, 1.96)	0.93 (0.46, 1.90)	0.94
**sEndoglin**	1.00 (Ref)	2.46 (0.98, 6.18)	1.83 (0.70, 4.77)	3.59 (1.47, 8.77)	0.004
**CRP**	1.00 (Ref)	1.09 (0.54, 2.20)	1.14 (0.56, 2.30)	1.11 (0.55, 2.23)	0.87
**CHI3L1**	1.00 (Ref)	2.02 (0.88, 4.64)	2.21 (0.92, 5.33)	2.36 (1.03, 5.33)	0.08
**C5a**	1.00 (Ref)	0.81 (0.38, 1.76)	1.33 (0.66, 2.68)	1.07 (0.53, 2.15)	0.69

^a^ Relative risks and 95% confidence intervals were estimated using binomial regression with the log link function. When the log-binomial model failed to converge, log-Poisson models were used.

^b^ Multivariate models adjusted for maternal age, marital status (yes/no), education (0–4, 5–7, 8–11, ≥ 12 years), Filmer-Pritchett wealth score less than median (yes/no), baseline gestational age, body mass index at baseline (lowest tertile vs. upper tertiles), frequency of meat consumption per week (once a week or less vs. more than once per week).

^c^ P-value for test for linear trend test calculated with median biomarker in each quartile as a continuous variable.

### Markers associated with sPTB in the training cohort were replicated in the test cohort

Based on the results of the training set we refined the biomarker panel to include 11 markers: Ang2, AngptL3, PGF, sTNFR2, sFlt-1, CHI3L1, C5a, sICAM-1, sEndoglin, IL18-BP and Leptin. We also included C5a, AngptL3 and sTNFR2 based on clinical and preclinical studies implicating these markers/pathways in pathophysiological pathways leading to sPTB [20, 23]. Similar to the training cohort, women in the test cohort who delivered sPTB had increased median levels of IL-18BP (*P* = 0.003), sICAM-1 (*P* = 0.01), sEndoglin (*P* = 0.03), CHI3L1 (*P* = 0.02) compared with women who delivered at term. Compared with term deliveries, in the test cohort we also observed increased median sTNFR2 (*P* = 0.01) and AngptL3 (*P* = 0.03) and reduced median Ang2 (*P* = 0.03) levels in women at 12–27 weeks gestation who subsequently delivered sPTB (Table 4). When we repeated the multivariate model in the test cohort we observed women with sICAM-1, sTNFR2, CHI3L1, C5a and IL18-BP in this highest quartile had an increased risk of delivering pre-term (p-value < 0.05 for linear trend test) compared to women in the lowest quartile. Women in the highest quartile for Ang-2 and Leptin had a reduced risk of PTB (p < 0.05, Table 5) compared to those in the lowest quartile. There was evidence of a linear trend in the associations between these biomarkers and sPTB (p-value <0.05 for linear trend test).

**Table 4:** Median biomarker^a^ values (pg/mL) according to term and preterm birth status in the testing cohort.

Biomarker^a^		Term Birth		Spontaneous PTB	
	n^b^	Median [IQR]	n^b^	Median [IQR]	P-Value^c^
**Ang-2**	523	4685.25 [2001.70, 9435.83]	105	4224.28 [1894.81, 8112.10]	0.03
**AngptL3**	518	70597.30 [44498.08, 104057.70]	104	77185.22 [42947.77, 121492.16]	0.03
**PGF**	495	900.01 [415.23, 1858.80]	99	975.95 [433.41, 1988.17]	0.14
**sFlt-1**	495	1260.93 [551.35, 2436.60]	99	1101.95 [489.52, 3341.08]	0.38
**sTNFR2**	520	4563.51 [2773.45, 6868.92]	105	5393.60 [3417.83, 8725.36]	0.01
**CHI3L1**	523	35167.74 [21489.03, 62798.91]	105	46241.21 [26809.22, 84268.91]	0.02
**C5a**	523	137880.60 [58478.32, 406637.08]	105	224461.74 [58232.14, 419980.47]	0.14
**sICAM-1**	523	143904.74 [95975.00, 209439.48]	105	164167.81 [106218.82, 237302.56]	0.01
**sEndoglin**	523	20053.81 [14098.36, 27112.65]	105	21942.55 [14371.43, 28020.64]	0.03
**IL-18BP**	523	12073.64 [8372.44, 18729.73]	105	14947.53 [9633.37, 22902.76]	0.003
**Leptin**	523	9092.89 [5005.73, 16282.79]	105	6970.72 [3777.01, 12217.92]	0.07

^a^Angiopoietin-2 (Ang2), Angiopoietin-Like 3 (AngptL3), Placental Growth Factor (PGF), Soluble fms-like tyrosine kinase 1 (sFlt-1), soluble Tumor Necrosis Factor Receptor 2 (sTNFR2), Chitinase-3-Like Protein-1 (CHI3L1), complement component C5a (C5a), soluble Intercellular Adhesion Molecule-1 (sICAM-1), soluble Endoglin (sEndoglin), Interleukin-18 Binding Protein (IL-18BP), and Leptin

^b^ Sample sizes were not equal for all biomarkers due to indeterminate assay results, where samples with undetectable values were excluded.

^c^ Results of Wilcoxon rank-sum test.

**Table 5:** Multivariate relative risks (95% Confidence Intervals) for sPTB according to quartiles in the testing cohort^a^ ^.^ ^b^.

	Q1	Q2	Q3	Q4	P-trend^c^
Ang-2	1.00 (Ref)	1.10 (0.70, 1.74)	0.87 (0.53, 1.41)	0.64 (0.37, 1.08)	**0.04**
AngptL3	1.00 (Ref)	0.79 (0.46, 1.35)	0.92 (0.57, 1.49)	1.44 (0.92, 2.25)	0.07
PGF	1.00 (Ref)	1.24 (0.73, 2.12)	1.37 (0.80, 2.35)	1.31 (0.78, 2.21)	0.43
sFlt-1	1.00 (Ref)	1.14 (0.71, 1.84)	0.69 (0.38, 1.18)	1.12 (0.54, 1.16)	0.76
sTNFR2	1.00 (Ref)	1.00 (0.58, 1.72)	1.57 (1.00, 2.56)	1.76 (1.09, 2.85)	**0.01**
CHI3L1	1.00 (Ref)	1.58 (0.94, 2.65)	1.34 (0.76, 2.38)	2.01 (1.22, 3.32)	**0.0017**
C5a	1.00 (Ref)	0.73 (0.42, 1.27)	1.17 (0.77, 1.79)	1.44 (0.81, 1.89)	0.02
sICAM-1	1.00 (Ref)	1.13 (0.68, 1.90)	1.37 (0.83, 2.26)	1.63 (1.01, 2.61)	**0.03**
sEndoglin	1.00 (Ref)	0.85 (0.50, 1.44)	1.24 (0.77, 2.01)	1.28 (0.81, 2.04)	0.17
IL-18BP	1.00 (Ref)	1.06 (0.59, 1.87)	1.72 (1.01, 2.92)	1.91 (1.17, 3.12)	**0.0014**
Leptin	1.00 (Ref)	0.85 (0.55, 1.32)	0.78 (0.50, 1.19)	0.39 (0.20, 0.73)	**0.0023**

^a^ Relative risks and 95% confidence intervals were estimated using binomial regression with the log link function. When the log-binomial model failed to converge, log-Poisson models were used.

^b^ Multivariate models adjusted for maternal age, marital status (yes/no), education (0–4, 5–7, 8–11, ≥ 12 years), Filmer-Pritchett wealth score less than median (yes/no), baseline gestational age, body mass index at baseline (lowest tertile vs. upper tertiles), frequency of meat consumption per week (once a week or less vs. more than once per week).

^c^ P-value for test of linear trend test calculated with median biomarker in each quartile as a continuous variable.

Based on the consistency of the results between the training and test cohorts we combined both cohorts for the subsequent analysis. In the combined cohort (n = 1060), women who went on to deliver sPTB (n = 156) has higher median levels of sTNFR2 (*P* = 0.01), CHI3L1 (*P* = 0.0005), C5a (*P* = 0.05), sICAM-1 (*P* = 0.01), sEndoglin (*P* = 0.0021), IL-18BP (*P* = 0.0005) and reduced median levels of Leptin (*P* = 0.0003) (S4 Table). Women with CHI3L1, C5a, sICAM-1, AngptL3, sEndoglin, sFlt-1 and IL-18BP in the highest quartile at enrollment had an increased relative risk of sPTB compared with women in the lowest quartile (S5 Table). Women in the highest quartile for Ang2 and Leptin had a 56% reduced risk of sPTB compared to those in the lowest quartile. Trend tests showed significant linear trends across quartiles of each biomarker and sPTB.

### Biomarker profiles at enrollment were differentially associated with early to moderate and late sPTB

To examine the associations between biomarkers and the degree of prematurity we examined the relative risk of early to moderate preterm (< 34 weeks gestation) and late preterm (34 to <37 weeks gestation) delivery in association with biomarkers. In the combined cohort, 61women delivered at <34 weeks and 95 women delivered between 34 and <37 weeks. Only 8 women delivered at <28 weeks and 17 women at <32 weeks, therefore we grouped extremely, very and moderate preterm deliveries together to allow for a sufficient sample size for analysis. Women in the early to moderate group had a mean gestational age at delivery of 32.3 [30.8, 33.3]. The relative risk of sPTB increased with increasing quartiles of CHI3L1, sICAM-1, and IL-18BP (Table 6, p-value < 0.05 for linear trend test); while the risk of late sPTB increased with increasing quartiles of sTNFR2, CHI3L1, C5a, sICAM-1 and IL-18BP. Leptin and Ang2 in the highest quartile were associated with a reduced relative risk of sPTB (Table 7) compared to the lowest quartile.

**Table 6:** Multivariate relative risks (95% Confidence Intervals) for early to moderate sPTB (< 34 weeks) in quartiles ^a^ ^,^ ^b^.

	Q1	Q2	Q3	Q4	P-trend^c^
Ang2	1.00 (Ref)	0.67 (0.31, 1.46)	0.56 (0.25, 1.25)	0.44 (0.18, 1.06)	0.10
AngptL3	1.00 (Ref)	1.50 (0.64, 3.50)	1.51 (0.60, 3.79)	2.22 (0.93, 3.79)	0.07
PGF	1.00 (Ref)	0.95 (0.42, 2.18)	1.03 (0.45, 2.39)	1.41 (0.65, 3.07)	0.17
sFlt-1	1.00 (Ref)	1.08 (0.71, 1.62)	1.25 (0.49, 3.208)	1.97 (0.83, 4.71)	0.11
sTNFR2	1.00 (Ref)	1.32 (0.55, 3.17)	1.63 (0.69, 3.86)	1.93 (0.85, 4.38)	0.47
CHI3L1	1.00 (Ref)	2.24 (0.81, 6.24)	3.02 (1.13, 8.05)	3.12 (1.17, 8.34)	**0.03**
C5a	1.00 (Ref)	2.18 (0.96, 4.94)	1.27 (0.49, 3.35)	1.97 (0.82, 4.76)	0.40
sICAM-1	1.00 (Ref)	0.78 (0.30, 2.04)	1.66 (0.71, 3.85)	2.06 (0.91, 4.68)	**0.024**
sEndoglin	1.00 (Ref)	1.22 (0.47, 3.11)	1.65 (0.69, 3.99)	1.95 (0.81, 4.74)	0.09
IL-18BP	1.00 (Ref)	0.83 (0.30, 2.33)	2.55 (1.15, 5.70)	1.90 (0.78, 4.62)	**0.043**
Leptin	1.00 (Ref)	0.25 (0.10, 0.66)	0.77 (0.40, 1.50)	0.54 (0.23, 1,24)	0.47

^a^ Relative risks and 95% confidence intervals were estimated using binomial regression with the log link function. When the log-binomial model failed to converge, log-Poisson models were used.

^b^ Multivariate models adjusted for maternal age, marital status (yes/no), education (0–4, 5–7, 8–11, ≥ 12 years), Filmer-Pritchett wealth score less than median (yes/no), baseline gestational age, body mass index at baseline (lowest tertile vs. upper tertiles), frequency of meat consumption per week (once a week or less vs. more than once per week).

^c^ P-value for test of linear trend test calculated with median biomarker in each quartile as a continuous variable.

**Table 7:** Multivariate relative risks (95% Confidence Intervals) for late sPTB (34 to < 37 weeks) according to quartiles^a^ ^,^ ^b^.

	Q1	Q2	Q3	Q4	P-trend^c^
Ang2	1.00 (Ref)	0.82 (0.50, 1.34)	0.79 (0.47, 1.32)	0.48 (0.27, 0.85)	**0.01**
AngptL3	1.00 (Ref)	0.94 (0.55, 1.60)	1.18 (0.69, 2.02)	1.34 (0.78, 2.31)	0.21
PGF	1.00 (Ref)	1.15 (0.66, 2.00)	1.34 (0.76, 2.36)	1.53 (0.90, 2.61)	0.19
sFlt-1	1.00 (Ref)	1.05 (0.60, 1.83)	0.83 (0.46, 1.50)	1.40 (0.85, 2.31)	0.11
sTNFR2	1.00 (Ref)	0.93 (0.51, 1.70)	1.47 (0.86, 2.51)	1.81 (1.07, 3.06)	**0.007**
CHI3L1	1.00 (Ref)	1.76 (0.94, 3.27)	1.47 (0.76, 2.84)	2.82 (1.56, 5.08)	**0.0002**
C5a	1.00 (Ref)	1.25 (0.71, 2.20)	1.25 (0.70, 2.25)	1.94 (1.15, 3.29)	**0.007**
sICAM-1	1.00 (Ref)	1.19 (0.68, 2.06)	0.98 (0.54, 1.79)	1.91 (1.12, 3.29)	**0.01**
sEndoglin	1.00 (Ref)	0.89 (0.50, 1.58)	1.25 (0.73, 2.11)	1.47 (0.88, 2.46)	0.07
IL-18BP	1.00 (Ref)	1.42 (0.77, 2.60)	1.76 (0.95, 3.18)	2.60 (1.47, 4.63)	**0.0003**
Leptin	1.00 (Ref)	1.34 (0.72, 1.80)	0.62 (0.36, 1.06)	0.55 (0.28, 1.07)	**0.03**

^a^ Relative risks and 95% confidence intervals were estimated using binomial regression with the log link function. When the log-binomial model failed to converge, log-Poisson models were used.

^b^ Multivariate models adjusted for maternal age, marital status (yes/no), education (0–4, 5–7, 8–11, ≥ 12 years), Filmer-Pritchett wealth score less than median (yes/no), baseline gestational age, body mass index at baseline (lowest tertile vs. upper tertiles), frequency of meat consumption per week (once a week or less vs. more than once per week).

^c^ P-value for test for linear trend test calculated with median biomarker in each quartile as a continuous variable.

### Biomarker profiles were associated with the gestational age at enrollment (time of plasma sample collection)

**Fig 1:**
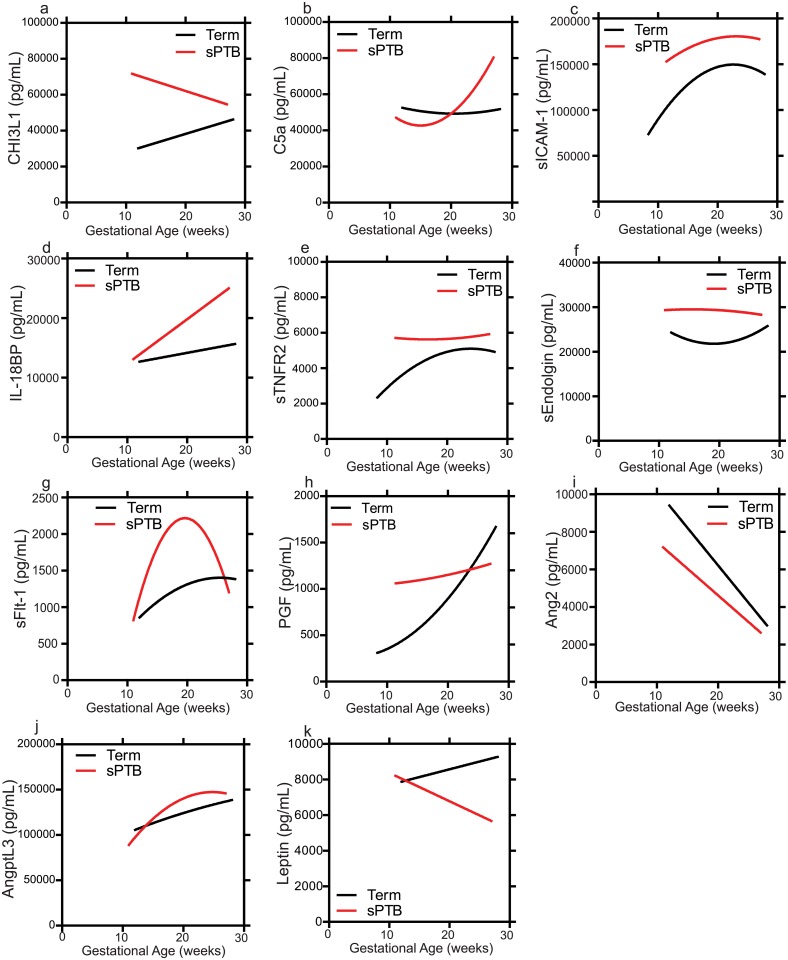
Levels of inflammatory mediators (a) CHI3L1, (b) C5a, (c) sICAM-1, (d) IL-18BP, (e) sTNFR2, (f) Endoglin and the angiogenic and anti-angiogenic mediators (g) sFlt-1, (h) PGF, (i) Ang2 and endocrine mediators (j) AngptL3 and (k) Leptin by gestational age at enrollment (time of blood sample collection) in term and spontaneous preterm births (sPTB). Figures depict polynomial curves with best fits to the data.

We observed informative profiles of biomarkers depending on the gestational age when samples were collected (< 20 weeks, 20–23 weeks, >23 weeks, Fig 1). Compared to women who went on to deliver full term, CHI3L1, sICAM-1, IL-18BP, and AngptL3 were elevated across gestational age at enrollment at all times under 23 weeks, whereas differences in the levels of Ang-2, C5a, sFlt-1, PGF and sTNFR2 between term and spontaneous preterm deliveries varied depending on the gestational age at which the sample was collected. For example compared to women who delivered full term, levels of C5a were not elevated early in gestation (<20 weeks) but were increased at later time points in gestation (<20 weeks) in women with sPTB. For multiple biomarkers (e.g. Ang2, sFlt-1, sTNFR2) samples collected at <20 weeks showed the strongest association with sPTB (Table 8). In samples collected between 20–23 weeks gestation women with levels in the highest quartiles PGF had an increased risk of delivering pre-term compared to women in the lowest quartile.

**Table 8:** Multivariate relative risks (95% Confidence Intervals) for sPTB according to quartiles by gestational age at plasma sample collection^a^ ^,^ ^b^.

		< 20 weeks	20–23 weeks	> 23 weeks
	No. of cases	85	48	29
Ang2	Q1	1.00 (Ref.)	1.00 (Ref.)	1.00 (Ref.)
	Q2	0.81 (0.49, 1.35)	1.42 (0.65, 3.10)	0.31 (0.09, 1.10)
	Q3	0.77 (0.44, 1.34)	1.17 (0.54, 2.51)	0.43 (0.15, 1.23)
	Q4	0.38 (0.21, 0.69)	1.21 (0.54, 2.69)	0.64 (0.22, 1.92)
	P-trend^c^	<0.0001	0.93	0.49
Angptl3	Q1	1.00 (Ref.)	1.00 (Ref.)	1.00 (Ref.)
	Q2	1.30 (0.73, 2.30)	1.48 (0.59, 3.68)	0.48 (0.15, 1.53)
	Q3	1.57 (0.86, 2.85)	2.21 (0.86, 5.15)	0.37 (0.12, 1.13)
	Q4	1.89 (1.06, 3.39)	2.13 (0.95, 4.76)	0.55 (0.20, 1.49)
	P-trend^c^	0.02	0.04	0.28
PGF	Q1	1.00 (Ref.)	1.00 (Ref.)	1.00 (Ref.)
	Q2	1.24 (0.69, 2.21)	1.01 (0.36, 2.84)	0.41 (0.15, 1.12)
	Q3	1.36 (0.72, 2.58)	1.88 (0.79, 4.50)	0.43 (0.16, 1.17)
	Q4	1.49 (0.86, 2.59)	2.21 (0.92, 5.30)	0.40 (0.13, 1.21)
	P-trend^c^	0.28	0.02	0.35
sFlt-1	Q1	1.00 (Ref.)	1.00 (Ref.)	1.00 (Ref.)
	Q2	1.32 (0.68, 2.56)	0.91 (0.40, 2.09)	1.21 (0.42, 3.49)
	Q3	0.99 (0.48, 2.05)	0.88 (0.42, 1.85)	1.05 (0.34, 3.21)
	Q4	1.86 (1.02, 3.39)	1.40 (0.74, 2.67)	0.76 (0.24, 2.41)
	P-trend^c^	0.02	0.21	0.53
sTNFR2	Q1	1.00 (Ref.)	1.00 (Ref.)	1.00 (Ref.)
	Q2	1.02 (0.54, 1.92)	1.32 (0.56, 3.11)	0.36 (0.10, 1.41)
	Q3	1.63 (0.94, 2.84)	1.13 (0.48, 2.69)	0.89 (0.32, 2.51)
	Q4	2.16 (1.23, 3.78)	1.40 (0.63, 3.11)	1.08 (0.39, 3.04)
	P-trend^c^	0.001	0.48	0.51
CHI3L1	Q1	1.00 (Ref.)	1.00 (Ref.)	1.00 (Ref.)
	Q2	2.40 (1.16, 4.96)	0.93 (0.35, 2.46)	1.82 (0.52, 6.35)
	Q3	2.28 (1.08, 4.83)	0.89 (0.35, 2.25)	2.16 (0.59, 7.96)
	Q4	3.23 (1.57, 6.63)	2.34 (1.08, 5.07)	1.52 (0.39, 5.91)
	P-trend^c^	0.0007	0.004	0.73
C5a	Q1	1.00 (Ref.)	1.00 (Ref.)	1.00 (Ref.)
	Q2	1.20 (0.71, 2.02)	2.00 (0.74, 5.38)	2.70 (0.58, 12.55)
	Q3	0.90 (0.49, 1.65)	1.50 (0.53, 4.25)	3.29 (0.64, 16.76)
	Q4	1.13 (0.67, 1.93)	3.13 (1.22, 8.00)	4.99 (1.06, 23.56)
	P-trend^c^	0.77	0.008	0.03
sICAM-1	Q1	1.00 (Ref.)	1.00 (Ref.)	1.00 (Ref.)
	Q2	1.51 (0.82, 2.78)	0.81 (0.32, 2.08)	0.43 (0.12, 1.53)
	Q3	1.34 (0.69, 2.59)	0.99 (0.41, 2.39)	0.90 (0.35, 2.31)
	Q4	2.42 (1.34, 4.40)	1.92 (0.90, 4.09)	0.56 (0.19, 1.63)
	P-trend^c^	0.002	0.03	0.49
sEndoglin	Q1	1.00 (Ref.)	1.00 (Ref.)	1.00 (Ref.)
	Q2	0.97 (0.53, 1.79)	1.63 (0.63, 4.18)	0.34 (0.07, 1.63)
	Q3	0.97 (0.53, 1.79)	2.05 (0.81, 5.17)	0.55 (0.15, 2.07)
	Q4	1.38 (0.78, 2.44)	1.62 (0.63, 4.19)	1.60 (0.60, 4.28)
	P-trend^c^	0.08	0.30	0.24
IL-18BP	Q1	1.00 (Ref.)	1.00 (Ref.)	1.00 (Ref.)
	Q2	1.33 (0.71, 2.49)	2.42 (0.6, 8.68)	0.26 (0.06, 1.21)
	Q3	1.94 (1.13, 3.35)	2.70 (0.79, 9.29)	0.70 (0.26, 1.88)
	Q4	1.93 (1.05, 3.55)	5.29 (1.60, 17.53)	0.72 (0.27, 1.92)
	P-trend^c^	0.01	0.0006	0.92
Leptin	Q1	1.00 (Ref.)	1.00 (Ref.)	1.00 (Ref.)
	Q2	0.83 (0.50, 1.40)	0.93 (0.48, 1.81)	0.92 (0.37, 2.35)
	Q3	0.72 (0.43, 1.21)	0.70 (0.32, 1.52)	0.43 (0.13, 1.45)
	Q4	0.69 (0.37, 1.27)	0.53 (0.20, 1.40)	0.28 (0.07, 1.18)
	P-trend^c^	0.25	0.20	0.06

^a^ Relative risks and 95% confidence intervals were estimated using binomial regression with the log link function. When the log-binomial model failed to converge, log-Poisson models were used.

^b^ Multivariate models adjusted for maternal age, marital status (yes/no), education (0–4, 5–7, 8–11, ≥ 12 years), Filmer-Pritchett wealth score less than median (yes/no), baseline gestational age, body mass index at baseline (lowest tertile vs. upper tertiles), frequency of meat consumption per week (once a week or less vs. more than once per week).

^c^ P-value for test for linear trend test calculated with median biomarker in each quartile as a continuous variable.

## Discussion

Little progress has been made in reducing sPTB in either resource-constrained or resource-rich settings. This is due, in part, to a limited understanding of the mechanisms underlying sPTB and the lack of tools that can be applied early in gestation to identify high-risk pregnancies. In this study we present data from a high disease burden setting supporting a role for altered angiogenic and inflammatory responses in the pathobiology of sPTB.

Based on the results of a training cohort we refined a panel of 11 markers and repeated our analysis on a testing cohort. In the test set we replicated the principal results of the training set observing elevated IL18-BP, sICAM-1, sEndoglin, CHI3L1and lower Ang2 and Leptin in sPTB cases. In the test set we also observed elevated sTNFR2, AngptL3 and C5a in women who delivered sPTB. With the combined training and test cohorts, we observed an association between biomarker levels with respect to timing of preterm birth (early/moderate [<34 weeks] versus late [>34-<37 weeks] preterm delivery) and gestational age at sample collection. Collectively these results suggest that levels of key angiogenic and inflammatory mediators measured in women when they typically first present for antenatal care in resource-constrained settings (~20 and 27 weeks gestation) may assist in identifying women at increased risk of sPTB.

The prospective design of the parent study, random selection of the nested cohorts, train and test structure, and the ability to control for multiple covariates are all strengths of the current study. A prevalence of 14.8 percent sPTB in this study is consistent with rates previously reported in Tanzania [2, 21]. To our knowledge this is the first study to assess mid-pregnancy biomarkers of sPTB in a low resource setting in Africa, where there is a very high burden of prematurity.

In this study gestational dating was performed by last menstrual period (LMP). While we recognize that the gold standard for gestational dating is ultrasound, this technology is often difficult to access in low resource settings. Younger maternal age, poor nutritional status, first pregnancy and fewer years of education are all associated with an increased risk of misclassification by LMP and were factors we included in our multivariate analysis [24]. In addition, post-term birth in this cohort could be a result of misclassification by LMP, which tends towards an overestimation of gestational age [24, 25]. We are now validating our findings with an external cohort of women using ultrasound dating. However, it is worth nothing that women in low resource settings typically make their first antenatal visit in the second trimester, when intrauterine growth restriction, which is common in these regions, can confound gestational dating by ultrasound. Moreover we feel it is important to conduct this research in the context of the standard of care in most resource poor regions, and the most feasible dating tool, which at this time is LMP.

Some studies have observed improved predictive value in combinations of several biomarkers compared with the use of markers individually [26–28]. A multi-marker predictive model developed based on this cohort will be validated on an external cohort in future studies. The results of this study will need to be tested in additional prospective studies with extended phenotyping of maternal complications, such as preeclampsia and gestational diabetes. Longitudinal mechanistic studies of pregnant women will also be required to determine the kinetics of these factors and how they vary over the course of normal and pathological pregnancies.

The results of this study add to the growing body of evidence implicating an imbalance of pro- and anti-angiogenic factors and altered host inflammatory responses in mediating adverse birth outcomes [10, 12, 18]. Although the molecular events leading to sPTB are not well understood, disruptions in early vascular development and placentation appear to influence the risk of sPTB outcomes [11]. Here we show, using a train and test approach as well as multivariate analysis, that elevated levels CHI3L1, IL-18BP, sICAM-1, sEndoglin, sTNFR2, AngptL3 and C5a as well as reduced Ang2 and Leptin were independent predictors of women who go on to deliver preterm.

Elevated pro-inflammatory markers in plasma, IL-18BP, sICAM-1, CHI3L1, C5a, and sTNFR2, were associated with an increased relative risk of sPTB. IL-18BP is a natural antagonist of the proinflammatory cytokine IL-18, and increases in response to elevated circulating levels of this cytokine. We used the levels of IL-18BP as a marker of an inflammatory response. CHI3L1 is a glycoprotein biomarker previously implicated in inflammatory responses as well as angiogenesis and extracellular matrix remodeling [29]. sICAM-1 is a soluble adhesion molecule cleaved in states of inflammation and endothelial activation [30]. Tumor necrosis factor (TNF) is a pro-inflammatory cytokine and increased levels of is soluble receptors, TNF receptor 1 and 2, have been observed in response to release of TNF [31]. Complement component C5a is a critical component of the complement cascade and a potent initiator and potent enhancer of pro-inflammatory and anti-angiogenic cascades [32]. Our results confirm an association between an elevated inflammatory response and spontaneous preterm delivery.

Numerous inflammatory proteins and receptors interact with angiogenic pathways. The initiation of pro-inflammatory pathways has been linked to generalized endothelial dysfunction [33]. Angiogenic mediators regulate the growth, structure and function of the placental vasculature, which impacts fetal growth and birth outcome. Elevated levels of anti-angiogenic sEndoglin and sFlt-1 were associated with an increased relative risk of sPTB while elevated levels of pro-angiogenic Ang2 were associated with a reduced risk. Soluble Endoglin, a soluble receptor of transforming growth factor (TGF) –β, is thought to play roles in immune regulation, angiogenesis and vascular integrity[34]. Soluble Endoglin also inhibits vascular permeability and nitric oxide-mediated vasodilation, which may alter placental vascularization and fetal growth. sEndgolin has previously been associated with sPTB [35, 36]. The angiopoietins (Ang1 and Ang2) competitively bind to sTie-2 receptor with Ang1 inducing vascular maturation and Ang2 antagonizing the effects of Ang1 and causing destabilization of the vascular network and angiogenesis [37]. Therefore the angiogenic profile associated with sPTB in this cohort may reflect a shift towards an anti-angiogenic profile in pregnancies at risk of sPTB.

Both Leptin and AngptL3 are associated with metabolic function. AngptL3 is a lipoprotein inhibitor with structural similarities to the angiopoietins that regulates the clearance of circulating lipids as well as playing a role in endothelial cell adhesion [38]. Leptin, a peptide hormone that regulates neuroendocrine function, also has inflammatory actions and has been implicated in adverse pregnancy outcomes, in particular preeclampsia [39, 40]. While both AngptL3 and Leptin have been linked with inflammatory and/or angiogenic pathways our results also suggest that disruptions in metabolic pathways are associated with sPTB.

There is evidence of a linear relationship between increased risk of mortality and morbidity as gestational age at delivery decreases [9]. Preterm delivery is now categorized as extremely preterm (<28 weeks), very preterm (28 to <32 weeks), moderate preterm (32 to <34 weeks) and late preterm (34 to <37 weeks) [4]. In this cohort the majority of deliveries at <34 weeks were moderate preterm. Eight women delivered at <28 weeks and 17 women delivered at <32 weeks which precluded us from examining extremely and very preterm birth cases independently. Elevated CHI3L1, sICAM-1 and IL-18BP were associated with an increased relative risk of sPTB in both the early to moderate and late preterm groups. Elevated sTNFR2, C5a, Ang2 and Leptin were associated with sPTB in only the late preterm group. These results suggest that there may be unique biomarker profiles associated with the degree of severity of preterm birth outcomes.

To date several studies have examined circulating maternal biomarkers of spontaneous preterm birth but few have reported individual markers with promising clinical utility and even fewer have been conducted in settings where the burden of sPTB is highest [41, 42]. The complex pathophysiology leading to sPTB likely results from the synergistic action of multiple physiological pathways. The tight regulation and temporal sequence of pro- and anti-angiogenic factors required for successful pregnancy outcomes suggests that angiogenic biomarker profiles measured early in pregnancy, and adjusted for gestational age, may help to risk-stratify subsequent birth phenotype. We observed an association between biomarkers and the gestational age at which the plasma sample was collected. Ang2, sFlt-1, and sTNFR2 were associated with sPTB only in samples collected at <20 weeks, whereas PGF was associated with sPTB in women with samples collected between 20–23 weeks gestation. In women with samples collected at >23 weeks C5a was associated with sPTB. In our cohort CHI3L1, IL-18BP and sICAM-1 were elevated at all time-points <23 weeks in those who went on to deliver sPTB. The results suggest that the levels and the corresponding balance between these pro- and anti-angiogenic factors will vary depending on the time of sampling, which was later in gestation in the current study.

In summary our data support a role for an early imbalance in angiogenic and inflammatory mediators in the pathobiology of sPTB and suggest potential biomarkers to risk-stratify women for sPTB and new targets for intervention to prevent this complication.

## Supporting Information

S1 Table Comparison of baseline characteristics between the first and second cohorts. (DOCX)

S2 Table Descriptive characteristics of the training cohort. Continuous data are presented as mean (STD) with t-test, categorical data are presented as n (%) with chi squared test.
(DOCX)

S3 Table Descriptive characteristics of the testing cohort. Continuous data are presented as mean (STD) with t-test, categorical data are presented as n (%) with chi squared test.
(DOCX)

S4 Table Median biomarker values (pg/mL) according to spontaneous preterm birth status in the combined training and test cohorts. Angiopoietin-2 (Ang2), Angiopoietin-Like 3 (AngptL3), Placental Growth Factor (PGF), Soluble fms-like tyrosine kinase 1 (sFlt-1), soluble Tumor Necrosis Factor Receptor 2 (sTNFR2), Chitinase-3-Like Protein-1 (CHI3L1), complement component C5a (C5a), soluble Intercellular Adhesion Molecule-1 (sICAM-1), soluble Endoglin (sEndoglin), Interleukin-18 Binding Protein (IL-18BP), and Leptin. Sample sizes were not equal for all biomarkers due to indeterminate assay results, where samples with undetectable values were excluded. Results of Wilcoxon rank-sum test.
(DOCX)

S5 Table Multivariate relative risks (95% Confidence Intervals) for sPTB according to quartiles in the combined training and test cohorts. Relative risks and 95% confidence intervals were estimated using binomial regression with the log link function. When the log-binomial model failed to converge, log-Poisson models were used. Multivariate models adjusted for maternal age, marital status (yes/no), education (0–4, 5–7, 8–11, ≥ 12 years), Filmer-Pritchett wealth score less than median (yes/no), baseline gestational age, body mass index at baseline (lowest tertile vs. upper tertiles), frequency of meat consumption per week (once a week or less vs. more than once per week). P-value for test for linear trend test calculated with median biomarker in each quartile as a continuous variable.
(DOCX)

## References

[1] Strategies GEGoCB, Constraints, George A, Young M, Bang A, Chan KY, et al Setting implementation research priorities to reduce preterm births and stillbirths at the community level. PLoS medicine. 2011;8(1):e1000380 10.1371/journal.pmed.1000380 21245907 PMC3014929

[2] Blencowe H, Cousens S, Oestergaard MZ, Chou D, Moller AB, Narwal R, et al National, regional, and worldwide estimates of preterm birth rates in the year 2010 with time trends since 1990 for selected countries: a systematic analysis and implications. Lancet. 2012;379(9832):2162–72. 10.1016/S0140-6736(12)60820-4 . 22682464

[3] Lozano R, Naghavi M, Foreman K, Lim S, Shibuya K, Aboyans V, et al Global and regional mortality from 235 causes of death for 20 age groups in 1990 and 2010: a systematic analysis for the Global Burden of Disease Study 2010. Lancet. 2013;380(9859):2095–128. 10.1016/S0140-6736(12)61728-0 . 23245604 PMC10790329

[4] WHO. Born Too Soon: The Global Action Report on Preterm Birth Geneva: March of Dimes, PMNCH, Save the Children, WHO, 2012

[5] Mwaniki MK, Atieno M, Lawn JE, Newton CR. Long-term neurodevelopmental outcomes after intrauterine and neonatal insults: a systematic review. Lancet. 2012;379(9814):445–52. 10.1016/S0140-6736(11)61577-8 22244654 PMC3273721

[6] Saigal S, Doyle LW. An overview of mortality and sequelae of preterm birth from infancy to adulthood. Lancet. 2008;371(9608):261–9. 10.1016/S0140-6736(08)60136-1 . 18207020

[7] Preterm Birth: Causes, Consequences and Prevention Washinton, D.C.: Institute of Medicine of the National Academies, 2007 20669423

[8] Goldenberg RL, Culhane JF, Iams JD, Romero R. Epidemiology and causes of preterm birth. Lancet. 2008;371(9606):75–84. 10.1016/S0140-6736(08)60074-4 . 18177778 PMC7134569

[9] Lawn JE, Gravett MG, Nunes TM, Rubens CE, Stanton C, Group GR. Global report on preterm birth and stillbirth (1 of 7): definitions, description of the burden and opportunities to improve data. BMC pregnancy and childbirth. 2010;10 Suppl 1:S1 10.1186/1471-2393-10-S1-S1 20233382 PMC2841772

[10] Andraweera PH, Dekker GA, Roberts CT. The vascular endothelial growth factor family in adverse pregnancy outcomes. Human reproduction update. 2012;18(4):436–57. 10.1093/humupd/dms011 . 22495259

[11] Silasi M, Cohen B, Karumanchi SA, Rana S. Abnormal placentation, angiogenic factors, and the pathogenesis of preeclampsia. Obstet Gynecol Clin North Am. 2010;37(2):239–53. 10.1016/j.ogc.2010.02.013 . 20685551

[12] Romero R, Gotsch F, Pineles B, Kusanovic JP. Inflammation in pregnancy: its roles in reproductive physiology, obstetrical complications, and fetal injury. Nutrition reviews. 2007;65(12 Pt 2):S194–202. . 18240548 10.1111/j.1753-4887.2007.tb00362.x

[13] Keelan JA, Blumenstein M, Helliwell RJ, Sato TA, Marvin KW, Mitchell MD. Cytokines, prostaglandins and parturition—a review. Placenta. 2003;24 Suppl A:S33–46. . 12842412 10.1053/plac.2002.0948

[14] Gibbs RS, Romero R, Hillier SL, Eschenbach DA, Sweet RL. A review of premature birth and subclinical infection. American journal of obstetrics and gynecology. 1992;166(5):1515–28. . 1595807 10.1016/0002-9378(92)91628-n

[15] Steinborn A, Niederhut A, Solbach C, Hildenbrand R, Sohn C, Kaufmann M. Cytokine release from placental endothelial cells, a process associated with preterm labour in the absence of intrauterine infection. Cytokine. 1999;11(1):66–73. 10.1006/cyto.1998.0399 . 10080881

[16] Romero R, Chaiworapongsa T, Erez O, Tarca AL, Gervasi MT, Kusanovic JP, et al An imbalance between angiogenic and anti-angiogenic factors precedes fetal death in a subset of patients: results of a longitudinal study. The journal of maternal-fetal & neonatal medicine: the official journal of the European Association of Perinatal Medicine, the Federation of Asia and Oceania Perinatal Societies, the International Society of Perinatal Obstet. 2010;23(12):1384–99. 10.3109/14767051003681121 20459337 PMC3023956

[17] Arias F, Victoria A, Cho K, Kraus F. Placental histology and clinical characteristics of patients with preterm premature rupture of membranes. Obstetrics and gynecology. 1997;89(2):265–71. 10.1016/S0029-7844(96)00451-6 . 9015033

[18] Kim YM, Bujold E, Chaiworapongsa T, Gomez R, Yoon BH, Thaler HT, et al Failure of physiologic transformation of the spiral arteries in patients with preterm labor and intact membranes. American journal of obstetrics and gynecology. 2003;189(4):1063–9. . 14586356 10.1067/s0002-9378(03)00838-x

[19] Levine RJ, Maynard SE, Qian C, Lim KH, England LJ, Yu KF, et al Circulating angiogenic factors and the risk of preeclampsia. The New England journal of medicine. 2004;350(7):672–83. 10.1056/NEJMoa031884 . 14764923

[20] Conroy AL, Silver KL, Zhong K, Rennie M, Ward P, Sarma JV, et al Complement activation and the resulting placental vascular insufficiency drives fetal growth restriction associated with placental malaria. Cell Host Microbe. 2013;13(2):215–26. 10.1016/j.chom.2013.01.010 . 23414761

[21] Fawzi WW, Msamanga GI, Urassa W, Hertzmark E, Petraro P, Willett WC, et al Vitamins and perinatal outcomes among HIV-negative women in Tanzania. The New England journal of medicine. 2007;356(14):1423–31. 10.1056/NEJMoa064868 . 17409323

[22] Silver KL, Zhong K, Leke RG, Taylor DW, Kain KC. Dysregulation of angiopoietins is associated with placental malaria and low birth weight. PloS one. 2010;5(3):e9481 Epub 2010/03/09. 10.1371/journal.pone.0009481 20208992 PMC2830425

[23] Thevenon AD, Zhou JA, Megnekou R, Ako S, Leke RG, Taylor DW. Elevated levels of soluble TNF receptors 1 and 2 correlate with Plasmodium falciparum parasitemia in pregnant women: potential markers for malaria-associated inflammation. Journal of immunology. 2010;185(11):7115–22. Epub 2010/10/29. doi: jimmunol.1002293 [pii] 10.4049/jimmunol.1002293 20980627 PMC2988086

[24] Hoffman CS, Messer LC, Mendola P, Savitz DA, Herring AH, Hartmann KE. Comparison of gestational age at birth based on last menstrual period and ultrasound during the first trimester. Paediatric and perinatal epidemiology. 2008;22(6):587–96. 10.1111/j.1365-3016.2008.00965.x . 19000297

[25] Savitz DA, Terry JW Jr., Dole N, Thorp JM Jr., Siega-Riz AM, Herring AH. Comparison of pregnancy dating by last menstrual period, ultrasound scanning, and their combination. American journal of obstetrics and gynecology. 2002;187(6):1660–6. . 12501080 10.1067/mob.2002.127601

[26] Goldenberg RL, Iams JD, Mercer BM, Meis PJ, Moawad A, Das A, et al The Preterm Prediction Study: toward a multiple-marker test for spontaneous preterm birth. American journal of obstetrics and gynecology. 2001;185(3):643–51. 10.1067/mob.2001.116752 . 11568793

[27] Schneuer FJ, Roberts CL, Ashton AW, Guilbert C, Tasevski V, Morris JM, et al Angiopoietin 1 and 2 serum concentrations in first trimester of pregnancy as biomarkers of adverse pregnancy outcomes. American journal of obstetrics and gynecology. 2014;210(4):345 e1–9. 10.1016/j.ajog.2013.11.012 . 24215861

[28] Bhat G, Williams SM, Saade GR, Menon R. Biomarker interactions are better predictors of spontaneous preterm birth. Reproductive sciences. 2014;21(3):340–50. 10.1177/1933719113497285 . 23885102

[29] Lee CG, Da Silva CA, Dela Cruz CS, Ahangari F, Ma B, Kang MJ, et al Role of chitin and chitinase/chitinase-like proteins in inflammation, tissue remodeling, and injury. Annual review of physiology. 2011;73:479–501. 10.1146/annurev-physiol-012110-142250 . 21054166 PMC3864643

[30] Krauss T, Emons G, Kuhn W, Augustin HG. Predictive value of routine circulating soluble endothelial cell adhesion molecule measurements during pregnancy. Clinical chemistry. 2002;48(9):1418–25. . 12194917

[31] Haider S, Knofler M. Human tumour necrosis factor: physiological and pathological roles in placenta and endometrium. Placenta. 2009;30(2):111–23. 10.1016/j.placenta.2008.10.012 19027157 PMC2974215

[32] Ward PA. The dark side of C5a in sepsis. Nature reviews Immunology. 2004;4(2):133–42. 10.1038/nri1269 . 15040586

[33] Molvarec A, Szarka A, Walentin S, Beko G, Karadi I, Prohaszka Z, et al Serum heat shock protein 70 levels in relation to circulating cytokines, chemokines, adhesion molecules and angiogenic factors in women with preeclampsia. Clinica chimica acta; international journal of clinical chemistry. 2011;412(21–22):1957–62. 10.1016/j.cca.2011.06.042 . 21756887

[34] ten Dijke P, Goumans MJ, Pardali E. Endoglin in angiogenesis and vascular diseases. Angiogenesis. 2008;11(1):79–89. 10.1007/s10456-008-9101-9 . 18283546

[35] Chaiworapongsa T, Romero R, Tarca A, Kusanovic JP, Mittal P, Kim SK, et al A subset of patients destined to develop spontaneous preterm labor has an abnormal angiogenic/anti-angiogenic profile in maternal plasma: evidence in support of pathophysiologic heterogeneity of preterm labor derived from a longitudinal study. The journal of maternal-fetal & neonatal medicine: the official journal of the European Association of Perinatal Medicine, the Federation of Asia and Oceania Perinatal Societies, the International Society of Perinatal Obstet. 2009;22(12):1122–39. 10.3109/14767050902994838 19916710 PMC3437777

[36] Kramer MS, Kahn SR, Platt RW, Genest J, Chen MF, Goulet L, et al Mid-trimester maternal plasma cytokines and CRP as predictors of spontaneous preterm birth. Cytokine. 2010;49(1):10–4. 10.1016/j.cyto.2009.08.014 . 19783155

[37] Charnock-Jones DS, Kaufmann P, Mayhew TM. Aspects of human fetoplacental vasculogenesis and angiogenesis. I. Molecular regulation. Placenta. 2004;25(2–3):103–13. 10.1016/j.placenta.2003.10.004 . 14972443

[38] Hato T, Tabata M, Oike Y. The role of angiopoietin-like proteins in angiogenesis and metabolism. Trends Cardiovasc Med. 2008;18(1):6–14. 10.1016/j.tcm.2007.10.003 . 18206803

[39] Mantzoros CS. Role of leptin in reproduction. Annals of the New York Academy of Sciences. 2000;900:174–83. . 10818404 10.1111/j.1749-6632.2000.tb06228.x

[40] Molvarec A, Szarka A, Walentin S, Beko G, Karadi I, Prohaszka Z, et al Serum leptin levels in relation to circulating cytokines, chemokines, adhesion molecules and angiogenic factors in normal pregnancy and preeclampsia. Reproductive biology and endocrinology: RB&E. 2011;9:124 10.1186/1477-7827-9-124 21906313 PMC3184629

[41] Conde-Agudelo A, Papageorghiou AT, Kennedy SH, Villar J. Novel biomarkers for predicting intrauterine growth restriction: a systematic review and meta-analysis. BJOG: an international journal of obstetrics and gynaecology. 2013;120(6):681–94. 10.1111/1471-0528.12172 . 23398929

[42] Menon R, Torloni MR, Voltolini C, Torricelli M, Merialdi M, Betran AP, et al Biomarkers of spontaneous preterm birth: an overview of the literature in the last four decades. Reproductive sciences. 2011;18(11):1046–70. 10.1177/1933719111415548 . 22031189

