# Supplementary material for: Inflammatory and Angiogenic Factors at Mid-Pregnancy Are Associated with Spontaneous Preterm Birth in a Cohort of Tanzanian Women
Source: PLoS One. 2015 Aug 6;10(8):e0134619. doi: 10.1371/journal.pone.0134619 (PMC4527774; doi:10.1371/journal.pone.0134619)
Supplement: S1 Table — (DOCX) [file pone.0134619.s001.docx]

|  | **Training Cohort** | **Test Cohort** | **p-value** |
| --- | --- | --- | --- |
| Mean gestational age at study entry | 21.26 | 21.21 | 0.83 |
| Mean age | 25.00 | 25.12 | 0.71 |
| Mean BMI | 24.89 | 24.97 | 0.76 |
| Mean baseline hemoglobin | 10.24 | 10.24 | 0.98 |
| Mean years of education | 7.15 | 7.00 | 0.35 |
| % Illiterate | 11.23 | 11.14 | 1.00 |
| % Married | 85.76 | 88.63 | 0.14 |
| % Per day spending for food (TShs) ≤ 500 | 46.90 | 48.33 | 0.70 |
| % District of recruitment |  |  |  |
| Ilala | 70.32 | 66.44 | 0.34 |
| Temeke | 10.05 | 10.42 |  |
| Kinondoni | 19.63 | 23.15 |  |

**S1 Table: Comparison of baseline characteristics between the first and second cohorts.**
