# Supplementary material for: Inflammatory and Angiogenic Factors at Mid-Pregnancy Are Associated with Spontaneous Preterm Birth in a Cohort of Tanzanian Women
Source: PLoS One. 2015 Aug 6;10(8):e0134619. doi: 10.1371/journal.pone.0134619 (PMC4527774; doi:10.1371/journal.pone.0134619)
Supplement: S2 Table — Continuous data are presented as mean (STD) with t-test, categorical data are presented as n (%) with chi squared test. (DOCX) [file pone.0134619.s002.docx]

**S2 Table: Descriptive characteristics of the training cohort**

|  |  | **Birth Outcome** | |  |
| --- | --- | --- | --- | --- |
| **Variable** |  | **Term (n=369)** | **Pre Term (n=57)** | **p-value** |
| **Gestational age at enrollment (weeks)** |  | 21.91 (3.34) | 19.29 (3.61) | <0.0001 |
| **Maternal age (years)** |  | 21.81 (3.07) | 20.92 (2.43) | 0.02 |
| **Education (years)** |  |  |  |  |
| 0-4 |  | 28 (7.59) | 1(1.75) | 0.03 |
| 5-7 |  | 237 (64.23) | 48 (84.21) |  |
| 8-11 |  | 89 (24.12) | 7 (12.28) |  |
| ≥ 12 |  | 15 (4.07) | 1 (1.75) |  |
| **Marital status** |  |  |  |  |
| Married |  | 294 (80.33) | 39 (72.58) | 0.07 |
| Divorced/single/widowed |  | 72 (19.67) | 17 (30.36) |  |
| **Filmer-Pritchett wealth score < median** |  |  |  |  |
| Yes |  | 207 (56.10) | 26 (45.61) | 0.25 |
| No |  | 162 (43.90) | 31 (54.39) |  |
| **Body Mass Index (kg/m^2^)** |  | 24.09 (3.36) | 22.76 (2.77) | 0.003 |
| **Baseline Hemoglobin (g/dL)** |  | 10.14 (1.39) | 9.66 (1.80) | 0.07 |
| **Baseline Skin-fold Thickness (cm)** |  | 17.84 (5.96) | 15.04 (3.86) | 0.0002 |
| **Birth Weight (g)** |  | 3079.3 (426.5) | 2823.2 (570.5) | 0.003 |
| **Peripheral Malaria Parasitaemia** |  |  |  |  |
| Yes |  | 4 (1.08) | 0 (0.00) | 0.43 |
| No |  | 365 (98.82) | 57 (100.00) |  |
| **Literacy** |  |  |  |  |
| Yes |  | 331 (90.19) | 52 (91.23) | 0.81 |
| No |  | 36 (9.81) | 5 (8.77) |  |
| **Frequency of meat/fish consumption** |  |  |  |  |
| ≤ 1x per week |  | 20 (5.42) | 6 (10.53) | 0.13 |
| > 1x per week |  | 349 (94.58) | 51 (89.47) |  |

Continuous data are presented as mean (STD) with t-test, categorical data are presented as n (%) with chi squared test.
