# Supplementary material for: Inflammatory and Angiogenic Factors at Mid-Pregnancy Are Associated with Spontaneous Preterm Birth in a Cohort of Tanzanian Women
Source: PLoS One. 2015 Aug 6;10(8):e0134619. doi: 10.1371/journal.pone.0134619 (PMC4527774; doi:10.1371/journal.pone.0134619)
Supplement: S3 Table — Continuous data are presented as mean (STD) with t-test, categorical data are presented as n (%) with chi squared test. (DOCX) [file pone.0134619.s003.docx]

**S3 Table: Descriptive characteristics of the testing cohort**

|  |  | **Birth Outcome** | |  |
| --- | --- | --- | --- | --- |
| **Variable** | **% Missing Data** | **Term (n=523)** | **Pre Term (n=105)** | **p-value** |
| **Gestational age at enrollment (weeks)** |  | 21.43 (3.5) | 19.84 (3.71) | <0.0001 |
| **Maternal age (years)** |  | 21.93 (3.17) | 21.55 (3.64) | 0.128 |
| **Education (years)** |  |  |  |  |
| 0-4 |  | 40 (7.5) | 15 (15) | 0.117 |
| 5-7 |  | 357 (67.0) | 62 (62) |  |
| 8-11 |  | 107 (20.1) | 19 (19) |  |
| ≥ 12 |  | 29 (5.4) | 4 (4) |  |
| **Marital status** |  |  |  |  |
| Married |  | 432 (79.4) | 73 (73) | 0.07 |
| Divorced/single/widowed |  | 110 (20.6) | 27 (27) |  |
| **Filmer-Pritchett wealth score < median** |  |  |  |  |
| Yes |  | 294 (55.5) | 53 (53) | 0.13 |
| No |  | 239 (44.8) | 47 (47) |  |
| **Body Mass Index (kg/m^2^)** |  | 23.70 (2.92) | 23.70 (2.93) | 0.93 |
| **Baseline Hemoglobin (g/dL)** |  | 10.2 (1.6) | 9.79 (1.69) | 0.008 |
| **Baseline Skin-fold Thickness (cm)** |  | 17.24 (6.03) | 16.21 (5.27) | 0.024 |
| **Birth Weight (g)** |  | 3105.92 (465.36) | 2823.66 (547.22) | 0.003 |
| **Peripheral Malaria Parasitaemia** |  |  |  |  |
| Yes |  | 2 (0.4) | 98 (98) | 0.06 |
| No |  | 531 (99.6) | 2 (2) |  |
| **Literacy** |  |  |  |  |
| Yes |  | 499 (91.6) | 88 (88) | 0.23 |
| No |  | 44 (8.3) | 12 (12) |  |
| **Frequency of meat/fish consumption** |  |  |  |  |
| ≤ 1x per week |  | 40 (7.5) | 6 (6) | 0.15 |
| > 1x per week |  | 493 (92.5) | 94 (91) |  |

Continuous data are presented as mean (STD) with t-test, categorical data are presented as n (%) with chi squared test.
