# Supplementary material for: Inflammatory and Angiogenic Factors at Mid-Pregnancy Are Associated with Spontaneous Preterm Birth in a Cohort of Tanzanian Women
Source: PLoS One. 2015 Aug 6;10(8):e0134619. doi: 10.1371/journal.pone.0134619 (PMC4527774; doi:10.1371/journal.pone.0134619)
Supplement: S4 Table — Angiopoietin-2 (Ang2), Angiopoietin-Like 3 (AngptL3), Placental Growth Factor (PGF), Soluble fms-like tyrosine kinase 1 (sFlt-1), soluble Tumor Necrosis Factor Receptor 2 (sTNFR2), Chitinase-3-Like Protein-1 (CHI3L1), complement component C5a (C5a), soluble Intercellular Adhesion Molecule-1 (sICAM-1), soluble Endoglin (sEndoglin), Interleukin-18 Binding Protein (IL-18BP), and Leptin. Sample sizes were not equal for all biomarkers due to indeterminate assay results, where samples with undetectable values were excluded. Results of Wilcoxon rank-sum test. (DOCX) [file pone.0134619.s004.docx]

**S4 Table: Median biomarker^a^ values (pg/mL) according to spontaneous preterm birth status in the combined** **training and test cohorts.**

| **Biomarkers** |  | **Term Birth** |  | **Preterm Birth** |  |
| --- | --- | --- | --- | --- | --- |
|  | **n^b^** | **Median [IQR]** | **n^b^** | **Median [IQR]** | **P-Value^c^** |
| **Ang2** | 891 | 4685.25 [2014.92, 9323.50] | 162 | 4224.28 [1780.21, 8137.86] | 0.08 |
| **AngptL3** | 887 | 87468.57 [56395.67, 131334.55] | 161 | 97238.70 [65956.22, 148633.56] | 0.23 |
| **PGF** | 845 | 1163.60 [534.43, 2163.68] | 154 | 1285.81 [589.66, 2809.59] | 0.27 |
| **sFlt-1** | 864 | 1211.60 [523.63, 2674.75] | 156 | 1574.30 [614.51, 3569.26] | 0.26 |
| **sTNFR2** | 889 | 4955.39 [3249.16, 7262.05] | 162 | 5779.06 [3770.88, 8776.04] | 0.01 |
| **CHI3L1** | 876 | 36529.76 [21631.72, 63504.14] | 162 | 51573.31 [27363.54, 86705.74] | 0.0005 |
| **C5a** | 880 | 73655.30 [29953.74, 213034.20] | 161 | 91468.80 [33550.15, 300472.95] | 0.05 |
| **sICAM-1** | 892 | 149462.17 [103426.26, 217211.15] | 162 | 179839.15 [115742.85, 256949.66] | 0.01 |
| **sEndoglin** | 891 | 20885.35 [14471.01,27806.71] | 162 | 22795.54 [16871.59, 430281.15] | 0.0021 |
| **IL-18BP** | 892 | 12483.14 [8598.85, 18215.01] | 162 | 15018.96 [10370.74, 24028.35] | 0.0005 |
| **Leptin** | 891 | 8653.17 [4776.67, 14456.89] | 162 | 6869.58 [3831.26, 11836.41] | 0.0003 |
|  |  |  |  |  |  |

^a^Angiopoietin-2 (Ang2), Angiopoietin-Like 3 (AngptL3), Placental Growth Factor (PGF), Soluble fms-like tyrosine kinase 1 (sFlt-1), soluble Tumor Necrosis Factor Receptor 2 (sTNFR2), Chitinase-3-Like Protein-1 (CHI3L1), complement component C5a (C5a), soluble Intercellular Adhesion Molecule-1 (sICAM-1), soluble Endoglin (sEndoglin), Interleukin-18 Binding Protein (IL-18BP), and Leptin

^b^ Sample sizes were not equal for all biomarkers due to indeterminate assay results, where samples with undetectable values were excluded.

^c^ Results of Wilcoxon rank-sum test.
