# Supplementary material for: Inflammatory and Angiogenic Factors at Mid-Pregnancy Are Associated with Spontaneous Preterm Birth in a Cohort of Tanzanian Women
Source: PLoS One. 2015 Aug 6;10(8):e0134619. doi: 10.1371/journal.pone.0134619 (PMC4527774; doi:10.1371/journal.pone.0134619)
Supplement: S5 Table — Relative risks and 95% confidence intervals were estimated using binomial regression with the log link function. When the log-binomial model failed to converge, log-Poisson models were used. Multivariate models adjusted for maternal age, marital status (yes/no), education (0–4, 5–7, 8–11, ≥ 12 years), Filmer-Pritchett wealth score less than median (yes/no), baseline gestational age, body mass index at baseline (lowest tertile vs. upper tertiles), frequency of meat consumption per week (once a week or less vs. more than once per week). P-value for test for linear trend test calculated with median biomarker in each quartile as a continuous variable. (DOCX) [file pone.0134619.s005.docx]

**S5 Table: Multivariate relative risks (95% Confidence Intervals) for sPTB according to quartiles in the combined training and test cohorts** **^a,b^**

|  | **Q1** | **Q2** | **Q3** | **Q4** | **P-trend**^c^ |
| --- | --- | --- | --- | --- | --- |
| **Ang2** | 1.00 (Ref) | 0.86 (0.60, 1.23) | 0.75 (0.51, 1.10) | 0.47 (0.31, 0.74) | 0.0006 |
| **AngptL3** | 1.00 (Ref) | 1.07 (0.71, 1.62) | 1.38 (0.93, 2.06) | 1.42 (0.95, 2.11) | 0.0397 |
| **PGF** | 1.00 (Ref) | 1.25 (0.82, 1.93) | 1.27 (0.83, 1.94) | 1.49 (1.01, 2.20) | 0.1034 |
| **sFlt-1** | 1.00 (Ref) | 1.08 (0.71, 1.62) | 0.88 (0.58, 1.35) | 1.44 (0.99, 2.08) | 0.0359 |
| **sTNFR2** | 1.00 (Ref) | 1.12 (0.72, 1.74) | 1.46 (0.99, 2.17) | 1.65 (1.12, 2.43) | 0.7600 |
| **CHI3L1** | 1.00 (Ref) | 1.80 (1.16, 2.80) | 1.74 (1.11, 2.74) | 2.47 (1.64, 3.74) | <0.0001 |
| **C5a** | 1.00 (Ref) | 1.30 (0.85, 1.98) | 1.05 (0.68, 1.62) | 1.76 (1.18, 2.63) | 0.0043 |
| **sICAM-1** | 1.00 (Ref) | 1.07 (0.70, 1.63) | 1.21 (0.80, 1.84) | 1.68 (1.15, 2.46) | 0.0025 |
| **sEndoglin** | 1.00 (Ref) | 0.96 (0.62, 1.50) | 1.47 (0.97, 2.23) | 1.45 (0.97, 2.16) | 0.0132 |
| **IL-18BP** | 1.00 (Ref) | 1.29 (0.82, 2.04) | 1.80 (1.17, 2.77) | 2.09 (1.40, 3.14) | <0.0001 |
| **Leptin** | 1.00 (Ref) | 0.86 (0.61, 1.21) | 0.72 (0.49, 1.06) | 0.56 (0.36, 0.89) | 0.0042 |

^a^ Relative risks and 95% confidence intervals were estimated using binomial regression with the log link function. When the log-binomial model failed to converge, log-Poisson models were used.

^b^ Multivariate models adjusted for maternal age, marital status (yes/no), education (0-4, 5-7, 8-11, ≥ 12 years), Filmer-Pritchett wealth score less than median (yes/no), baseline gestational age, body mass index at baseline (lowest tertile vs. upper tertiles), frequency of meat consumption per week (once a week or less vs. more than once per week)

^c^ P-value for test for linear trend test calculated with median biomarker in each quartile as a continuous variable.
